# miR-1 Inhibits Cell Growth, Migration, and Invasion by Targeting VEGFA in Osteosarcoma Cells

**DOI:** 10.1155/2016/7068986

**Published:** 2016-09-29

**Authors:** Junjie Niu, Yibao Sun, Qiaoge Guo, Dongju Niu, Bo Liu

**Affiliations:** ^1^Department of Radiology, Zhengzhou Orthopedics Hospital, Zhengzhou, China; ^2^Department of Minimally Invasive Spine Surgery, Zhengzhou Orthopedics Hospital, Zhengzhou, China; ^3^Zhengzhou Key Laboratory of Bone and Joint Imaging, Zhengzhou, China; ^4^Department of Radiology, Zhengzhou Second Hospital, Zhengzhou, China; ^5^Department of Function, Zhengzhou Orthopedics Hospital, Zhengzhou, China

## Abstract

microRNAs (miRNAs) are small noncoding RNAs and have been shown to play a crucial role in the osteosarcoma (OS) tumorigenesis and progression. VEGFA is a key regulator of angiogenesis and plays an important role in regulation of tumor metastasis. The objective of this study was to determine whether VEGFA was involved in miR-1-mediated suppression of proliferation, migration, and invasion of OS cells. The expression levels of miR-1 were significantly lower in OS tumor tissues than those in adjacent normal tissues and in SAOS-2 and U2OS cell lines compared to a normal osteoblast (NHOst) cell line. VEGFA was upregulated in OS tumor tissues and SAOS-2 and U2OS cell lines. The results of CCK-8 assay and transwell assay showed that miR-1 acted as a tumor suppressor by inhibiting cell proliferation, migration, and invasion in U2OS cells. Dual luciferase reporter assay demonstrated that VEGFA was a direct and functional target gene of miR-1. miR-1 directly inhibits the protein expression of VEGFA via its 3′-UTR. Knockdown of VEGFA by siRNA inhibited proliferation, migration, and invasion of U2OS cells. Our study suggested the potential inhibitory function of miR-1 in OS cell proliferation, migration, and invasion via inhibiting VEGFA.

## 1. Introduction

Osteosarcoma (OS) is the most common primary malignancy that occurs in bone, especially in children and young adults [1]. The majority of the patients with OS died of pulmonary metastasis after surgical treatment. The 5-year survival rate of patients with no metastatic disease is 60%–70%, but patients with metastatic disease present a lower 5-year survival rate of less than 30%. Through the application of neoadjuvant chemotherapy, the 5-year survival rate of OS patients has increased to approximately 50%–80% [2]. In recent years, microRNAs (miRNAs) have been reported to play vital roles in the progression of a large variety of human cancers [3]. However, due to the high level of chromosomal instability and the complex karyotypes, roles of miRNAs in the molecular pathogenesis and etiology of OS are still not fully elucidated.

miRNAs are a class of short noncoding endogenous RNAs of 19–24 nucleotides in length and act as a regulator in a variety of cellular activities including cell proliferation, differentiation, apoptosis, immune activation, angiogenesis, and tumorigenesis [4]. miRNAs are capable of regulating the expression of protein-coding genes via interacting with their 3′-untranslated regions (UTR), resulting in either mRNA cleavage or interruption of protein synthesis [5]. A growing body of evidence has indicated that miR-1 is associated with cell proliferation, migration, and invasion in many kinds of cancers and functions as a tumor suppressor with high potential to diminish tumor development [6, 7]. A comparative analysis of miRNA expression profile confirmed the 5-fold decrease of miR-1 and miR-133b in OS tumors in comparison with paired normal tissues [8]. Real-time polymerase chain reaction (RT-PCR) in 56 patients with OS showed that the expression levels of miR-1, miR-133b, and miR-378^*∗*^ in tumors were significantly decreased compared with the normal bone from noncancer patients. Moreover, this study also provided an evidence that the miR-1 mRNA level was lower in 31 high-grade OS than that in 25 low-grade OS and in metastatic tumors as compared to nonmetastatic tumors [9].

Angiogenesis is a crucial determinant in tumor initiation, progression, and metastasis. The vascular endothelial growth factor A (VEGFA) protein is a chemical signaling molecule that plays a central role in physiological and tumor-induced angiogenesis and is also a target of antiangiogenic therapies [10]. Tumor cells produce VEGFA protein to promote the development of vasculature, providing a sufficient supply of oxygen and nutrients, suggesting that VEGFA could be a potential target for cancer therapy [11]. Overexpression of VEGFA has been detected in a number of human tumor tissues. Moreover increased VEGFA gene expression has been found frequently to be associated with tumor progression, recurrences, and the 5-year survival rate of patients that suffered from malignancy [12]. A large number of researches have been done showing that miRNAs play important roles in vascular development and angiogenesis [13]. miR-1 and miR-206 negatively regulate developmental angiogenesis by directly reducing the expression level of VEGFA in muscle and endothelium during zebrafish development, identifying the crucial function of miRNAs on regulation of cross-tissue signaling [14].

In this study, we determined the expression levels of miR-1 in 34 paired OS tumor tissues and OS cell lines. Furthermore, we investigated the inhibitory function of miR-1 on VEGFA protein expression and explored the regulatory roles of miR-1 in proliferation, migration, and invasion of OS cells* in vitro*.

## 2. Materials and Methods

### 2.1. Patients and Tissue Samples

**Figure 1:**
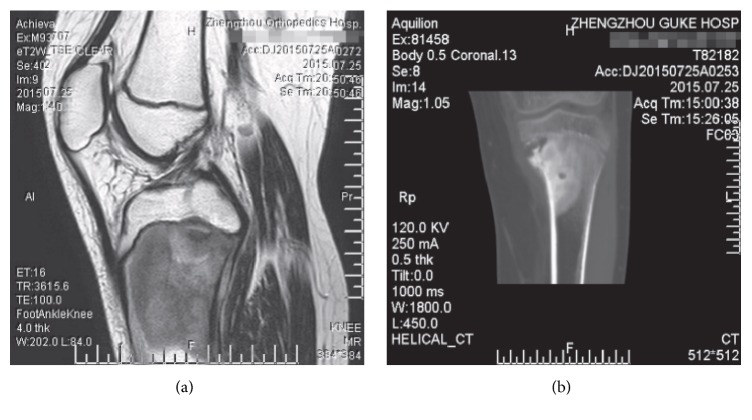
The imaging features of OS. (a) MRI findings included osteolytic destruction, joint effusion, and soft tissue mass. (b) CT analysis showed osteolytic bone destruction in proximal tibia.

Primary OS tumor tissue samples were gained from 34 patients with OS (Figures 1(a) and 1(b)) in Zhengzhou Orthopedics Hospital between August 2012 and November 2014. The patients were diagnosed with OS according to the criteria of the World Health Organization. Neither chemotherapy nor radiotherapy had been used in all of the patients before surgical resection of the primary tumor. Tumor stage was assessed in accordance with the tumor-lymph node-metastasis classification system of the International Union against Cancer. Thirty-four tumor samples and paired adjacent noncancerous tissues were removed and then immediately frozen and stored in liquid nitrogen for further RT-PCR assay. This study was approved by the Ethics Review Board at Zhengzhou Orthopedics Hospital. Tissue samples were collected after obtaining written informed consents from each participant.

### 2.2. Cell Culture and Transfection

OS cell lines (American Type Culture Collection, Manassas, VA, USA) were maintained in Dulbecco modified Eagle medium (DMEM, Gibco, Carlsbad, CA, USA) supplemented with 10% fetal bovine serum (Gibco), 100 U/mL penicillin (Sigma-Aldrich, Louis, MO, USA), and 100 *μ*g/mL streptomycin (Sigma-Aldrich) in a humid incubator (Forma Scientific, Marietta, OH, USA) with 95% O_2_ and 5% CO_2_ at 37°C. OS cells were transfected 24 h after being seeded in 6-well plate at a concentration of 5 × 10^5^ cells. VEGFA siRNA, scramble siRNA, miR-1 mimic (100 nM), and control miRNA mimic (GenePharm, Shanghai, China) were transfected into OS cells using Lipofectamine 2000 transfection reagent (Invitrogen, Carlsbad, CA, USA) according to the manufacturer's instructions. After 6 h, the culture medium was replaced with 2 mL DMEM containing 10% FBS and antibiotics. Cells were harvested for gene expression analysis 48 h after transfection.

### 2.3. RNA Isolation and Quantitative Real-Time PCR

Total RNA was isolated using TRIzol reagent (Invitrogen) according to the manufacturer's protocol. RNA purity and concentrations were detected on a Nanodrop 2000 (Thermo Fisher Scientific). RNA integrity was assessed on an agarose gel with ethidium bromide staining by electrophoresis. The total RNA was reverse-transcribed into cDNA using the Reverse Transcription Kit (Invitrogen) according to the manufacturer's protocol. The miR-1 expression level was quantified using SYBR green qRT-PCR assay (Invitrogen). Specific primers (Sangon Biotech, Shanghai, China) were designed as follows: miR-1 forward: 5′-TCA ATC TCT AAC AAG CTA ATC TCT-3′; miR-1 reverse: 5′-TTG ACA GTA GGT TAA TCC AAA GT-3′; U6 forward, 5′-AAC GCT TCA CGA ATT TGC GT-3′; U6 reverse, 5′-CTC GCT TCG GCA GCA CA-3′. Data were analyzed according to the 2^−ΔΔCt^ cycle threshold method and normalized to nuclear RNA U6 as an endogenous control for the expression of miRNA-1.

### 2.4. Luciferase Reporter Assay

The dual luciferase reporter plasmid fused with the wild type or mutant 3′-UTR segment of human VEGFA was purchased from GenePharma, Shanghai, China. For verification of the regulation of VEGFA by miR-1, approximately 1 × 10^5^ cells per well were cultured in a 24-well plate one day before transfection so that they reach 70–80% confluence in the day of transfection. The next day, cells were cotransfected with 100 ng wild type or mutant VEGFA 3′-UTR luciferase reporter plasmid and 50 nM miR-1 mimic or scramble mimic using Lipofectamine 2000 (Invitrogen). After 48 h, cells were harvested using passive lysis buffer (Promega, Madison, WI, USA) and the luciferase activity was measured by the Dual-Luciferase Reporter Assay System (Promega) according to the manufacturer's instructions.

### 2.5. Western Blot Analysis

Cells were harvested 48 h after transfection and then total proteins were extracted by RIPA lysis buffer (Sigma-Aldrich) and protein concentration was determined by BCA protein assay (Pierce, Rockford, IL, USA). Protein samples were separated by sodium dodecyl-sulfate polyacrylamide gel electrophoresis and then transferred to a polyvinylidene difluoride membrane (PVDF, Bio-Rad, Hercules, CA, USA). The PVDF membrane was blocked in 5% (w/v) nonfatty milk powder in Tris-buffered saline (TBS, 10 mm Tris, and 100 mm NaCl) containing 0.1% Tween-20 (Sigma-Aldrich) for 1 h and then washed with TBS containing 0.05% Tween-20 (TBST) to remove redundant milk. The PVDF membranes were probed with a primary antibody such as anti-VEGFA antibody (Cell Signaling Technology, Danvers, MA, USA) or anti-GAPDH antibody (Santa Cruz Biotechnology, Santa Cruz, USA) overnight at 4°C. The PVDF membrane was washed with TBST three times (15 min each). PVDF membranes were incubated with the corresponding secondary antibodies (Invitrogen) for 1 h at room temperature. After washing three times (20 min each) with TBST at room temperature, the membranes were immersed in chemiluminescence detection reagent (Pierce) for 3 min and then exposed to X-ray film for 5–10 s.

### 2.6. Cell Viability Assay

Cell viability was assessed using the Cell Counting Kit-8 (CCK-8, Dojindo Molecular Technologies, Rockville, MD) assays according to the manufacturer's instructions. U2OS cells were seeded into 96-well plates at a density of 5 × 10^3^ cells/well. The next day, cells were transfected with miR-1 mimic or scramble mimic. At 0, 24, 48, and 72 h after transfection, 10 *μ*L CCK-8 mixture solution was added to each well, and then the plates were incubated at 37°C in a 5% CO_2_ incubator for 1 to 1.5 h until visual color conversion occurred. The absorbance in each well at 450 nm was measured with a microplate reader (Biotek Synergy HT, Winooski, VT, USA).

### 2.7. Cell Migration and Invasion Assay

The upper chamber of the basal membrane was coated with 5 *μ*g/*μ*L Matrigel (BD Biosciences, Franklin Lakes, NJ, USA). Transwell filters (pore size, 8 *μ*m, Corning Incorporated, Corning, NY, USA) were placed in 24-well plates. At 24 h after transfection, cells were added to the compartment of a transwell chamber containing DMEM with 0.1% bovine serum albumin. DMEM containing 10% fetal bovine serum was used as the chemoattractant. After 24 h at 37°C, cells on the upper membrane surface of the filter were gently wiped off with a cotton swab, and the filters were fixed with 95% ethanol for 30 minutes and then stained with 0.2% crystal violet solution for 30 minutes. Invasion cells adhering to the undersurface were fixed and stained with 0.1% crystal violet. The total number of invading cells was counted in five representative fields under microscopy (200x magnification).

### 2.8. Statistical Analysis

All experiments were conducted with at least three replicates and the data are expressed as the mean ± standard error of the mean. Comparison between two groups was made using Student's* t*-test. All statistical analysis was performed using SPSS 16.0 software (SPSS Inc., Chicago, IL, USA). Statistical significance in every figure was represented as follows: ^*∗*^ *P* < 0.05 and ^*∗∗*^ *P* < 0.01; NS, not significant. A value of *P* < 0.05 was considered significant.

## 3. Results

### 3.1. miR-1 Expression Level Was Downregulated and VEGFA Was Upregulated in OS Samples and Cells

**Figure 2:**
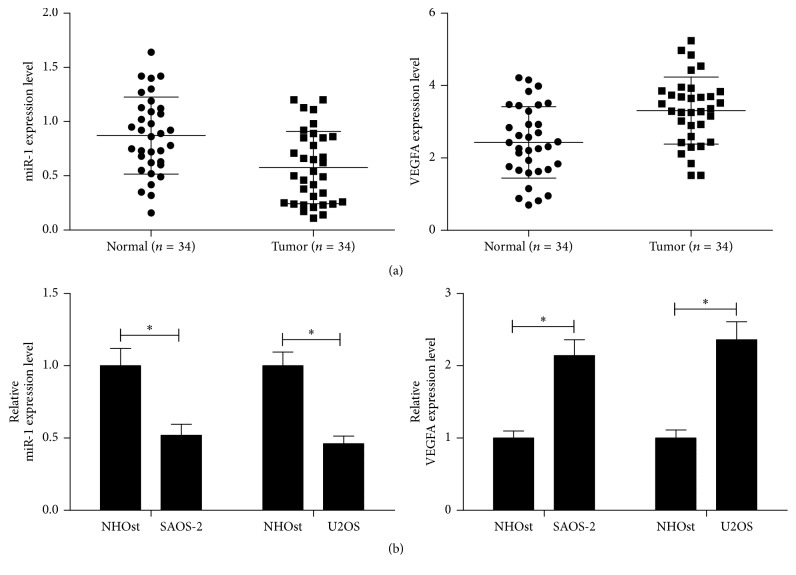
The effect of miR-1 and VEGFA aberrant expression in OS samples and cells. The expression levels of miR-1 and VEGFA in tumors from patients with OS (a) and in OS cell lines (SAOS-2 and U2OS, (b)) were measured using qRT-PCR. Data were expressed as the mean ± SD (*n* = 3). ^*∗*^ *P* < 0.01.

Previous studies have shown that miR-1 expression was strongly downregulated in OS cell lines relative to normal osteoblast (NHOst) cell line. However, its exact role in OS has not been verified as yet. To confirm the result, qRT-PCR was conducted in 34 paired OS samples and OS cells. As shown in Figure 2, miR-1 was significantly decreased in OS tissues (Figure 2(a)) as well as in SAOS-2 and U2OS cells (Figure 2(b)), but the expression level of VEGFA was increased.

### 3.2. miR-1 Suppresses Growth, Migration, and Invasion of U2OS Cells

**Figure 3:**
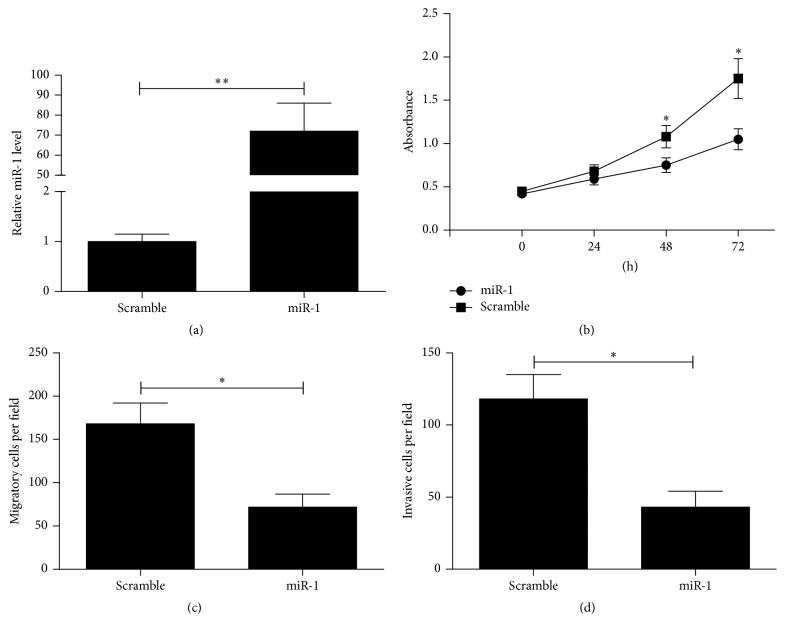
The effect of miR-1 on proliferation, migration, and invasion of U2OS cells. (a) CCK-8 assays for U2OS cells were done to assess the inhibitory effects of miR-1 on cell proliferation. (b, c) Cell migration and invasion assays of U2OS cells were done to evaluate the effects of miR-1 on cell migration and invasion. Data were expressed as the mean ± SD (*n* = 3). ^*∗*^ *P* < 0.01, ^*∗∗*^ *P* < 0.001.

To explore further the effects of miR-1 on OS cells, U2OS cells were treated with miR-1 mimic or scramble mimic. After transfection with miR-1 mimic, the intracellular level of miR-1 was approximately 70-fold higher in U2OS cells when compared to the scramble control group (Figure 3(a)). The effect of miR-1 on cell growth was evaluated using the CCK-8 assay. We found that treatment with miR-1 significantly suppressed cell growth in U2OS cells (Figure 3(b)).

Cell migration and invasion were known to promote tumor metastasis, which is the main cause of death in patients with OS. Thus, we explored the effects of miR-1 on migration and invasion of U2OS cells using transwell assays with or without Matrigel. Transwell assays without Matrigel showed that miR-1 overexpression in U2OS cells resulted in a significant reduction in the number of cells passing through the chambers compared with the scramble group (Figure 3(c)). Before the invasion assays, the chambers were coated with Matrigel. As shown in Figure 3(d), the number of cells passing through the chambers in the miR-1 group was significantly smaller than that in the miR-control group. These data indicate that miR-1 suppresses cell migration and invasion of OS cells* in vitro*.

### 3.3. VEGFA Is a Putative Target Gene of miR-1 in U2OS Cells

**Figure 4:**
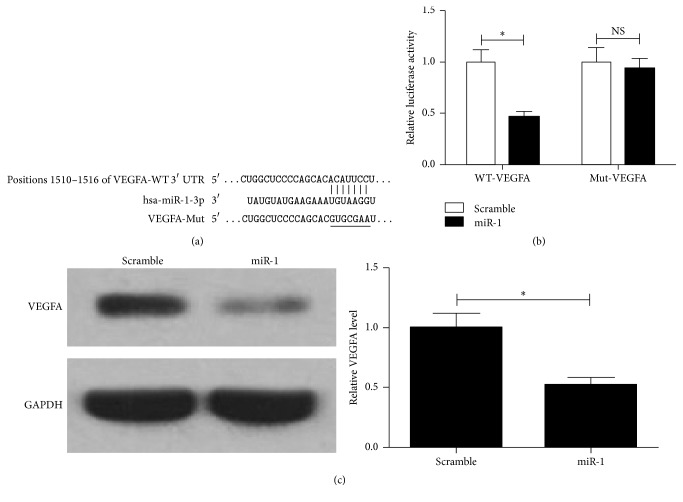
The interaction between miR-1 and VEGFA. (a) Putative binding sites for miR-1 on VEGFA 3′-UTR were shown. The mutated region in VEGFA 3′-UTR was labeled by a horizontal line. (b) U2OS cells were cotransfected with a luciferase reporter containing the wild type (WT) or mutant (Mut) VEGFA 3′-UTR, along with miR-1 mimic or scramble mimic. The relative luciferase activities were measured 24 h after transfection. (c) The protein expression level of VEGFA was detected by western blot. A representative western blot and quantitative results of densitometry are shown. Data were expressed as the mean ± SD (*n* = 3). ^*∗*^ *P* < 0.01; NS, no significant difference compared to control.

To assess whether miR-1 directly regulates VEGFA expression by binding the 3′-UTR of VEGFA mRNA in U2OS cells, luciferase reporter vector containing the putative VEGFA 3′-UTR target site for miR-1 and the mutant version were cotransfected with miR-1 mimic or scramble mimic into U2OS cells. The predicted miR-1-binding sites in human VEGFA 3′-UTR and the mutant sites of VEGFA 3′-UTR were labeled in Figure 4(a). Luciferase reporter assay showed that overexpression of miR-1 significantly inhibited the luciferase activity of VEGFA with the wild type 3′-UTR, but the luciferase activity of VEGFA with the mutant 3′-UTR was not changed (Figure 4(b)). The result of western blot showed that the expression of VEGFA significantly decreased in miR-1 mimic group compared with that in scramble mimic group (Figure 4(c)). Taken together, these results demonstrated that miR-1 directly inhibits VEGFA expression at posttranscriptional level through targeting its 3′-UTR region.

### 3.4. Knockdown of VEGFA Inhibits Growth, Migration, and Invasion of U2OS Cells

**Figure 5:**
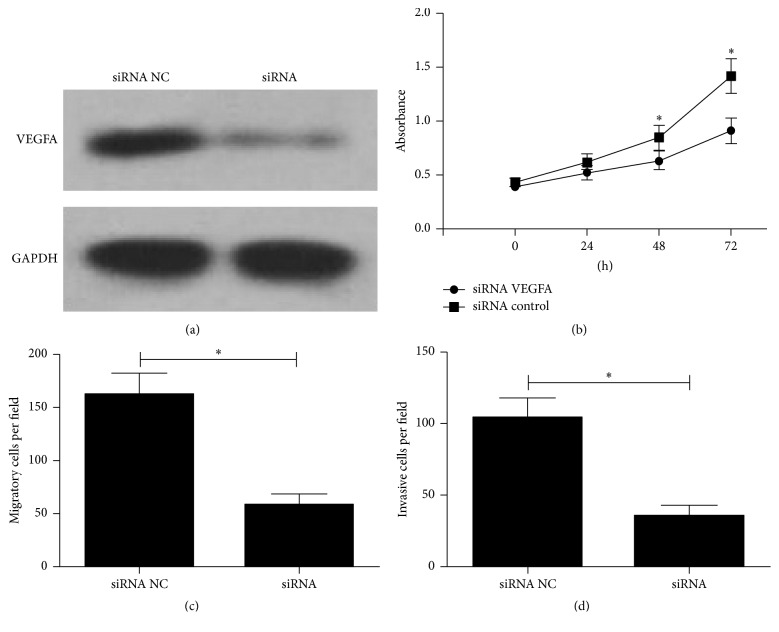
The effect of VEGFA knockdown on proliferation, migration, and invasion of U2OS cells. (a) Western Blot was performed to detect the expression levels of VEGFA in U2OS cells transfected with VEGFA siRNA or control siRNA. The inhibitory effects of miR-1 on cell proliferation (b), cell migration (c), and invasion (d) were assessed after transfection with VEGFA siRNA or control siRNA. Data were represented as the mean ± SD (*n* = 3). ^*∗*^ *P* < 0.01.

In order to test whether the inhibitory effects of miR-1 on the growth, migration, and invasion of U2OS cells are mediated by VEGFA, endogenous VEGFA gene in U2OS cells was knocked down by RNA interference method. siRNA-mediated suppression of VEGFA was confirmed by western blot assay. As shown in Figure 5(a), the expression level of VEGFA protein was significantly decreased in the VEGFA siRNA group compared with that in the siRNA control group. On the other hand, downregulation of VEGFA significantly inhibited cell growth, migration, and invasion in U2OS cells (Figures 5(b)–5(d)). These data indicated that was a key mediator in miR-1-mediated suppression of growth, migration, and invasion in OS cells.

## 4. Discussion

miRNAs mediate posttranscriptional regulation of genes by inhibiting translation or destabilizing target mRNAs. Numerous miRNAs have been demonstrated to be as oncogenes or tumor suppressors regulating the expression of cancer-related genes. An increasing number of studies have identified the miRNAs that were frequently aberrant in tumor tissues compared with normal control. Several miRNAs, such as miR-140, miR-34, miR-29b, and miR-422a, have been proved to be implicated in tumorigenesis, tumor progression, and prognosis of the patients with OS [15–17]. Therefore, clarifying the exact mechanisms underlying the roles of disease-associated miRNAs in OS pathological process may provide new opportunities for diagnosis and therapy in the future. Our study showed that the expression of miR-1 was downregulated in OS cells, and miR-1 mimic suppressed proliferation, migration, and invasion of OS cells* in vitro*. Furthermore, VEGFA was identified to be a target gene of miR-1 in OS cells.

miR-1, as a tumor suppressor in many types of cancers, is downregulated in human primary cancer tissues and cell lines. For example, Jiang et al. reported that miR-1 was downregulated and inhibited cell proliferation of esophageal squamous cell carcinoma* in vivo* and* in vitro* by downregulating MET, cyclin D1, and CDK4 [18]. Chen et al. showed that miR-1 suppressed cell growth and metastasis of nasopharyngeal carcinoma cells by targeting K-ras [19]. Minemura et al. found that abnormal expression of miR-1 was related to an aggressive phenotype of breast carcinoma and was regarded as a potent prognostic factor in breast carcinoma [20]. Previous studies have suggested that exogenous miR-1 is capable of suppressing the expression of protooncogenes, such as c-MET (receptor tyrosine kinase) and Pim-1 (serine/threonine kinase) [21]. miR-1, abundant in the cardiac and smooth muscles, was significantly decreased in tumor tissues from patients with OS when compared with the paired normal bones [22]. Therefore, miR-1 may be involved in the pathogenesis of OS. Epigallocatechin-3-gallate (EGCG), a polyphenolic compound from green tea, has been proved to have anticancer capability in various human cancers [23]. miR-1 mimic treatment enhanced EGCG-induced inhibition on cell proliferation of OS cells. A previous study demonstrated that miR-1 mimic suppressed c-MET expression, and combination treatment with EGCG and c-MET inhibitor (crizotinib) inhibited the growth rates of OS cells. Their results suggested that EGCG exhibited an anticancer function* in vitro* and* in vivo*, at least partially, via regulating miR-1/c-MET interaction [24]. In this study, the result of qRT-PCR showed that the expression level of miR-1 was decreased in clinical OS tumor tissues. We demonstrated that miR-1-mediated downregulation of VEGFA inhibited cell growth, migration, and invasion in OS cells, suggesting that miR-1 might act as a tumor suppressor in OS at least partially through targeting VEGFA.

VEGFA is thought to be the primary stimulator of angiogenesis, during the course of development and in a variety of pathological conditions [25]. VEGFA stimulates vascular endothelial cells by binding tyrosine kinase receptors to VEGF receptors 1 and 2 (VEGFR1 and VEGFR2). During the early hypoxic stage of cancer cells, VEGFA is responsible for cell proliferation, production of angiogenic factors, and formation of new endothelial cells so as to meet the demand of vasculogenesis [26], suggesting that VEGFA is a vital stimulator required for tumor growth and metastasis, including osteosarcoma. For example, Zhuang and Wei reported that the meta-analysis suggested that the expression of VEGF was closely related to poorer overall survival in patients with osteosarcoma [27]. Lammli et al. showed that high expression of VEGF induced a higher recurrence and more frequent cases of remote metastasis in OS [28]. During developmental angiogenesis, VEGF acts as an essential component of the angiogenic response of various kinds of tumors, which makes it an ideal clinical target for the treatment of cancer, including OS [29]. A growing number of researches have suggested that miRNAs regulated OS cell growth, invasion, and migration by targeting VEGFA. For example, Shao et al. found that mir-497 downregulation promoted cell growth and cisplatin resistance in OS cells by targeting VEGFA through PI3K/Akt pathway [30]. Zhang et al. showed that miR-29b negatively regulated VEGF expression and overexpression of miR-29b inhibited cell proliferation, migration, and invasion of OS MG63 cells by binding to VEGF [31]. Several experiments have been done to assess the effect of ectopic miR-1 expression on OS cell behavior in U2-OS cell lines, suggesting that miR-1 reduced proliferative capacity of OS cells and led to transient perturbations in cell cycle [9, 32]. However, the exact molecular mechanism underlying the regulatory effect of miRNAs on OS cell behavior is not well known. In order to investigate the putative mechanism underlying the role of miR-1 in tumor angiogenesis, a dual luciferase experiment was performed for identification of the target of this miRNA. Finally, VEGFA was confirmed as a target of miR-1 in U2OS cells. Moreover, VEGFA knockdown attenuated the effective inhibition of miR-1 mimic on cell growth, migration, and invasion of U2OS cells. Therefore, VEGFA may be required for the function of miR-1 in inhibiting tumor growth and metastasis.

The present study found a downregulation of miR-1 in OS clinical specimens and highlighted the function of miR-1 in the control of OS cell behavior. miR-1 inhibited cell growth, migration, and invasion by targeting VEGFA in OS cells. The identification of the miR-1-regulated VEGFA pathway provided a new insight into the potential molecular mechanisms of OS. Further experiments should be carried out to study the anticancer role of miR-1* in vivo* and to investigate the roles of other aberrant miRNAs and their target proteins as potential prognostic and therapeutic indicators.
